# Targeted Biomarker Profiling of Matched Primary and Metastatic Estrogen Receptor Positive Breast Cancers

**DOI:** 10.1371/journal.pone.0088401

**Published:** 2014-02-10

**Authors:** Erica B. Schleifman, Rupal Desai, Jill M. Spoerke, Yuanyuan Xiao, Cheryl Wong, Ilma Abbas, Carol O’Brien, Rajesh Patel, Teiko Sumiyoshi, Ling Fu, Rachel N. Tam, Hartmut Koeppen, Timothy R. Wilson, Rajiv Raja, Garret M. Hampton, Mark R. Lackner

**Affiliations:** 1 Department of Oncology Biomarker Development, Genentech Inc., South San Francisco, California, United States of America; 2 Department of Biostatistics, Genentech Inc., South San Francisco, California, United States of America; 3 Department of Research Pathology, Genentech Inc., South San Francisco, California, United States of America; Health Canada and University of Ottawa, Canada

## Abstract

Patients with newly diagnosed, early stage estrogen receptor positive (ER+) breast cancer often show disease free survival in excess of five years following surgery and systemic adjuvant therapy. An important question is whether diagnostic tumor tissue from the primary lesion offers an accurate molecular portrait of the cancer post recurrence and thus may be used for predictive diagnostic purposes for patients with relapsed, metastatic disease. As the class I phosphatidylinositol 3' kinase (PI3K) pathway is frequently activated in ER+ breast cancer and has been linked to acquired resistance to hormonal therapy, we hypothesized pathway status could evolve over time and treatment. Biomarker analyses were conducted on matched, asynchronous primary and metastatic tumors from 77 patients with ER+ breast cancer. We examined whether *PIK3CA* and *AKT1* alterations or PTEN and Ki67 levels showed differences between primary and metastatic samples. We also sought to look more broadly at gene expression markers reflective of proliferation, molecular subtype, and key receptors and signaling pathways using an mRNA analysis platform developed on the Fluidigm BioMark™ microfluidics system to measure the relative expression of 90 breast cancer related genes in formalin-fixed paraffin-embedded (FFPE) tissue. Application of this panel of biomarker assays to matched tumor pairs showed a high concordance between primary and metastatic tissue, with generally few changes in mutation status, proliferative markers, or gene expression between matched samples. The collection of assays described here has been optimized for FFPE tissue and may have utility in exploratory analyses to identify patient subsets responsive to targeted therapies.

## Introduction

Clinical management of breast cancer is currently based on diagnostic evaluation of expression of estrogen receptor (ER), progesterone receptor (PR) and HER2. Such analyses are typically conducted on primary tumor tissue collected at the time of diagnosis, although many patients will survive for years without local or distant disease recurrence. Moreover, a variety of studies have shown discordance in ER status between primary and metastatic samples ranging from 10 to 40% [1], [2], [3]. This has sometimes been taken to reflect a true switch in biology of the tumor in response to therapy, but has also been attributed to sampling error in focally-receptor positive disease and limited accuracy and reproducibility of the assays for receptor expression [3].

Given that novel targeted therapies are being developed in ER+ breast cancer, changes in the biology of the tumor that occur during adjuvant therapy could adversely impact predictive value of diagnostic assessments conducted on primary tumor samples but used to guide therapy in metastatic patients. In particular, the phosphatidylinositol 3' kinase (PI3K)/mTOR pathway has been linked in a variety of studies to acquired resistance to endocrine therapies both preclinically and clinically [4], and recent clinical results have validated this idea by showing that the mTOR inhibitor everolimus in combination with the aromatase inhibitor exemestane extends survival in patients with metastatic ER+ breast cancer who have progressed on prior endocrine therapy [5]. Upregulation or mutational activation of this pathway during adjuvant or front-line metastatic therapy could confound interpretation of predictive biomarkers conducted on primary tumor samples.

To address the question of whether primary and metastatic ER+ breast cancer samples are generally similar in terms of biomarker prevalence, and hence whether primary tissue is an accurate indicator of biomarker status in later stage patients, we evaluated a panel of asynchronously collected matched primary and metastatic tumors with a panel of biomarkers related to proliferation, epithelial-mesenchymal biology and PI3K pathway signaling. Previous studies have found that such tissues are generally concordant for *PIK3CA* and PTEN status [6], [7]. In addition, gene expression profiling of eight matched primary and metastatic breast cancer samples of mixed subtype has shown that gene expression profiles of primary breast tumors are generally maintained in distant metastases [8]. Here we extend these findings by showing in a large collection of ER+ breast tumors that gene expression profiles, as well as proliferation status, remain remarkably similar despite intervening time and treatment with both hormonal and chemotherapy regimens. Specifically the proliferation marker Ki67 showed a Pearson correlation coefficient of 0.76 between matched primary and metastatic samples, *PIK3CA*, *AKT1* and PTEN status were all at least 90% concordant, and only six out of 90 genes showed a statistically significant difference in mRNA expression between primary versus metastatic tissue. Thus, archival primary tumor tissue provides a surprisingly accurate portrait of biomarker status in patients with disease recurrence. The collection of assays described in this manuscript has been extensively optimized and validated for low quantity, fixed archival tissue, suggesting it may provide a useful paradigm for generating exploratory biomarker data that can be used to understand therapeutic response and resistance in the context of clinical trials.

## Materials and Methods

### Tumor specimens and cell lines

Formalin-fixed paraffin-embedded (FFPE) tumor blocks were obtained for 77 patients with paired asynchronously collected matched primary and metastatic tumors. Tissue samples were obtained from Cureline, Inc (South San Francisco, CA) following approval of the Ethics Committee of Saint Petersburg City Clinical Oncology Hospital and appropriate confirmation of written informed consent. Tissue samples were also obtained from The MT Group (Van Nuys, CA) following IRB approval (http://www.sterlingirb.com). The IRB waived the need for written informed consent per FDA guidelines, as this was a retrospective study with anonymized patient data. Hematoxylin-eosin (H&E) sections were prepared for all samples and were reviewed by a pathologist to confirm diagnosis and assess tumor content. ER, PR and HER2 status was determined by local testing. Breast cancer cell lines used in this study were obtained from the American Type Culture Collection (ATCC, Manassas, VA) or from the Deutsche Sammlung von Mikroorganismen und Zellkulturen GmbH (DSMZ, Braunschweig, Germany). Cell lines were archived at an early passage in the Genentech cell bank and authenticated either by a multiplex short tandem repeat assay or as previously described [9], [10].

### Immunohistochemistry (IHC)

PTEN IHC was conducted on FFPE tumor samples using the Discovery XT automated staining platform (Ventana, Tucson, AZ). Sections were subjected to antigen retrieval with Cell Conditioning I (CC1, Ventana). The primary antibody was obtained from Cell Signaling Technologies (clone 138G6, Danvers, MA). Reactions were developed using the UltraMap DAB detection system (Ventana), and counterstained with Hematoxylin II (Ventana). PTEN was scored in a semiquantitative fashion by a single pathologist using an H-score method to account for heterogeneity of expression. The percentage of tumor cells present at each of four staining intensities was scored, and the H-score was calculated as follows:

H  =  (3 × % of strongly stained cells) + (2 × % of moderately stained cells) + (1 × % of weakly stained cells) + (0 × % of cells without staining). This gave a score ranging from total absence of PTEN in the tumor compartment (H-score 0) to PTEN expression in tumor cells equivalent to surrounding normal and stromal cells (H-score 300).

Ki67 IHC was conducted on FFPE tumor samples using the Leica Bond-III automated slide stainer (Leica Microsystems, Buffalo Grove, IL). Sections were subjected to antigen retrieval with Target Retrieval Solution (Dako, Carpinteria, CA). The primary antibody was obtained from NeoMarkers/LabVision (clone SP6, Fremont, CA). Reactions were developed using the Vectastain Elite ABC- Peroxidase detection system (Vector Labs, Burlingame, CA), and counterstained with Mayer’s Hematoxylin. The percentage of tumor cells demonstrating nuclear expression of Ki67 was assessed by a single pathologist.

### DNA and RNA isolation from FFPE tumor tissue

Five slides of FFPE tumor sections per sample were macrodissected to enrich for neoplastic tissue, as identified by pathologist assessment. The slides were then deparaffinized using three five minute incubations in Envirene followed by a three minute incubation in 100% ethanol and then a two minute incubation in 100% ethanol. All slides were then dried for five to ten minutes before the tissue was placed into a microcentrifuge tube containing tumor lysis buffer and Proteinase K. Tubes were incubated at 55°C for three to 16hrs to allow for complete digestion and release of nucleic acids. Lysates were then aliquoted and store at −80°C until isolation was performed. RNA was isolated using the High Pure FFPE RNA Micro Kit (Roche Applied Sciences, Indianapolis, IN) according to the manufacturer’s protocol. DNA was isolated using the QIAamp DNA FFPE Tissue kit according to the manufacturer’s protocol (Qiagen, Hilden, Germany). RNA and DNA were stored at −80°C until analyses were performed.

### Mutation and Gene Expression Analysis

Genomic DNA was analyzed for mutations in *AKT1* and *PIK3CA* as described previously [11]. RNA (10−100 ng) was subjected to a one-step cDNA synthesis/preamplification reaction using the Invitrogen Platinum Taq/Reverse Transcriptase enzyme mix as per the manufacturer’s protocol with the exception that PCR cycling conditions were changed from a 14 cycle preamplification to 18 cycles (Life Technologies, Carlsbad, CA). Following amplification, samples were diluted one to four with TE and qPCR was conducted on Fluidigm 96.96 Dynamic Arrays using the BioMark™ HD system according to the manufacturer’s protocol and as previously described [12]. Samples were run in triplicate and cycle threshold (Ct) values were converted to relative expression values (negative delta Ct) by subtracting the mean of the six reference genes from the mean of each target gene. Hierarchical clustering was carried out on median-centered data with the complete linkage method using Cluster v3.0 and visualized using Treeview [13].

### SNP analysis

Cases where primary and metastatic samples were discordant for *PIK3CA*, PTEN and *AKT1* were verified to be from the same patient by SNP genotyping. Genomic DNA (25−100 ng) was preamplified with primer sets for 48 unique human SNPs for 14 cycles and analyzed using the Fluidigm SNPtype™ assay platform according to the manufacturer’s protocol (Fluidigm Corporation, South San Francisco, CA). Data was analyzed in the automated genotype calling algorithm using the Fluidigm SNP Genotyping Software (v3.1.1).

### Statistical Analysis

Statistical analysis was performed using the R statistical software [14]. Differences in *PIK3CA* and *AKT1* mutation and PTEN loss frequencies between paired primary and metastatic tumor samples was assessed using McNemar tests. Fluidigm raw Ct data were normalized by applying median normalization. Differential expression analysis was carried out employing Limma’s empirical Bayes moderated statistics [15]. Genes that were differentially expressed between primary and metastatic samples were identified using paired t statistics. Similarly, gene expression differences between the three subtypes were assessed using ANOVA models. P-values were adjusted for multiple testing errors using the Benjamini-Hochberg false discovery rate.

## Results

### PI3KCA mutation and PTEN prevalence and overlap in ER+ breast cancer

**Figure 1:**
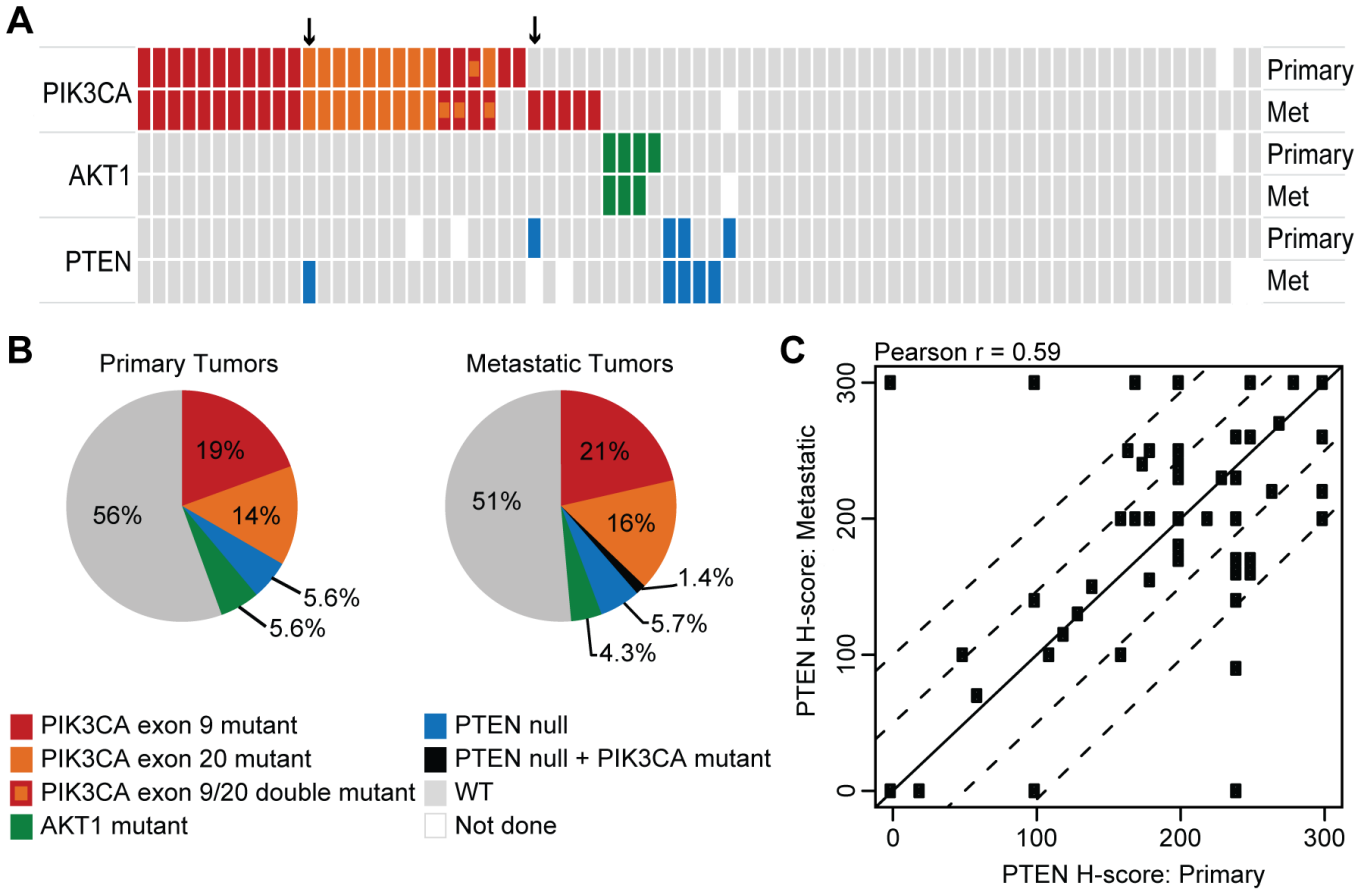
PI3K pathway alterations in primary and metastatic ER+ breast cancers. (A) Distribution of alterations in *PIK3CA*, *AKT1* and PTEN across 75 matched primary and metastatic ER+ breast cancers. PTEN null status denotes total absence of PTEN protein in neoplastic cells determined by immunohistochemistry. Arrows indicate patients with alterations in both *PIK3CA* and PTEN. (B) Frequency and overlap of PI3K pathway alterations in ER+ breast cancer samples. Biomarker frequencies calculated only from patients where tissue was evaluable for all biomarker assays. The data from the single *PIK3CA* exon 4 mutant sample was pooled with the exon 9 data, and the data from the exon 9/20 double mutant samples were pooled with exon 20 data. (C) Scatterplot of PTEN protein levels indicated by H-score in primary and metastatic samples. The solid diagonal line (y = x) and the dashed lines (y = x±50, y = x±100) are shown to highlight the magnitude of the absolute differences between x and y axes.

A collection of FFPE ER+ breast tumors representing matched primary and metastatic samples (Table S1) were obtained to determine the prevalence and overlap of a panel of candidate biomarkers. Patients received a wide variety of hormonal and chemotherapy regimens in the adjuvant and front-line metastatic settings, and the metastatic samples were all collected at least six months after the primary sample, with a mean of 31 months and median of 19 months (Table S1). DNA extracted from each tumor pair was used to determine *PIK3CA* and *AKT1* mutation status with a sensitive and specific qPCR-based assay [11], and serial sections from each tumor pair were also used to assess Ki67 staining and total PTEN loss via IHC (Table 1). *PIK3CA* mutation status was 90% concordant between primary and metastatic tumors (McNemar *P* = 0.45), with a prevalence of 33% and 38%, respectively (Figure 1A and B, Table 1). One patient had two distinct hotspot mutations present in the primary tumor, while three patients showed two different mutations in the metastatic lesion (Figure 1A, Table 1). *AKT1* mutation status was 99% concordant between primary and metastatic tumors (McNemar *P* = 0.48), with a prevalence of 5.6% and 4.3%, respectively. PTEN status, when scored as presence or complete absence of staining by IHC, was 93% concordant between primary and metastatic tumors (McNemar *P* = 0.62), with a prevalence of 5.6% and 7.1%, respectively. Intermediate levels of PTEN staining were also evaluated using an H-score, and were found to show moderate to high correlation between primary and metastatic samples (Pearson *r* = 0.58, Figure 1C). In the case of all three biomarkers, examples of presence in the primary and absence in the metastatic sample were apparent, suggesting that the discordance cannot simply be explained by the acquisition of the alteration over time and after treatment. In addition, alterations in *PIK3CA*, *AKT1* and PTEN were generally non-overlapping (Figure 1A and B, Table 1), with two exceptions. In one case, a patient with *PIK3CA* mutations in both primary and metastatic samples showed low PTEN expression in the primary tumor and was PTEN null in the metastatic sample (Figure 1A, Table 1). In a second case, a patient with a *PIK3CA* mutation in the metastatic sample showed PTEN loss in the primary tumor, though the metastatic sample was not evaluable for PTEN (Figure 1A, Table 1). Overall 44% of primary and 49% of metastatic samples showed evidence of PI3K pathway activation based on analysis of these three markers (Figure 1B).

**Table 1:** PIK3CA, AKT1, PTEN and Ki67 status across a collection of 77 matched ER+ breast cancers.

	PIK3CA status		AKT1 status		PTEN H-Score		Ki67 % positive	
Patient Number	Primary tumor	Metastatic tumor	Primary	Metastatic	Primary	Metastatic	Primary	Metastatic
HP-53153	**E542K**	**E542K**	MND	MND	130	130	35	40
HP-56947	**E542K**	**E542K**	MND	MND	300	220	15	10
HP-53151	**E542K**	**E542K**	MND	MND	230	230	45	30
HP-53107	**E542K**	**E542K**	MND	MND	165	250	25	20
HP-53115	**E542K**	**E542K**	MND	MND	300	260	10	10
HP-53143	**E542K**	**E542K**	MND	MND	270	270	30	30
HP-53079	**E542K**	**E542K**	MND	MND	300	300	10	25
HP-50681	**E545K**	**E545K**	MND	MND	200	180	20	5
HP-53065	**E545K**	**E545K**	MND	MND	160	200	40	70
HP-53103	**E545K**	**E545K**	MND	MND	200	230	30	50
HP-56290	**E545K**	**E545K**	MND	MND	300	200	10	NA
HP-53097	**H1047L**	**H1047L**	MND	MND	20	**0**	30	40
HP-53073	**H1047R**	**H1047R**	MND	MND	180	155	20	15
HP-53095	**H1047R**	**H1047R**	MND	MND	220	200	75	75
HP-53077	**H1047R**	**H1047R**	MND	MND	230	230	20	15
HP-53101	**H1047R**	**H1047R**	MND	MND	250	260	25	15
HP-53061	**H1047R**	**H1047R**	MND	MND	250	300	80	80
HP-49238	**H1047R**	**H1047R**	MND	MND	240	160	20	25
HP-50663	**H1047R**	**H1047R**	MND	MND	NA	300	30	80
HP-56292	**H1047R**	**H1047R**	MND	MND	160	100	75	85
HP-51208	**E545K**	**E545K/H1047R**	MND	MND	300	300	25	NA
HP-56953	**E545K**	**H1047R/E545X**	MND	MND	NA	130	10	10
HP-53063	**E545K/H1047R**	**E545K**	MND	MND	50	100	75	80
HP-56949	**H1047R**	**H1047R/E545K**	MND	MND	240	200	90	85
HP-53147	**E542K**	MND	MND	MND	265	220	30	20
HP-49251	**N345K**	MND	MND	MND	200	300	15	2
HP-49235	MND	**E545K**	MND	MND	**0**	NA	10	7
HP-51382	MND	**E545K**	MND	MND	230	230	40	35
HP-49257	MND	**E542K**	MND	MND	200	NA	NA	15
HP-53111	MND	**Q546X**	MND	MND	250	170	15	5
HP-51712	MND	**E542K**	MND	MND	180	250	10	10
HP-53119	MND	MND	**E17K**	**E17K**	240	230	15	10
HP-53085	MND	MND	**E17K**	**E17K**	200	250	15	60
HP-49242	MND	MND	**E17K**	**E17K**	300	300	30	40
HP-53121	MND	MND	**E17K**	MND	200	200	25	60
HP-53089	MND	MND	MND	MND	**0**	**0**	40	40
HP-50665	MND	MND	MND	MND	**0**	**0**	60	80
HP-53059	MND	MND	MND	MND	100	**0**	30	30
HP-49249	MND	MND	MND	MND	240	**0**	20	50
HP-49240	MND	NA	MND	NA	**0**	300	15	5
HP-49247	MND	MND	MND	MND	300	300	60	25
HP-56957	MND	MND	MND	MND	60	70	40	15
HP-56959	MND	MND	MND	MND	100	Positive	20	10
HP-56300	MND	MND	MND	MND	100	300	NA	60
HP-51386	MND	MND	MND	MND	100	140	10	30
HP-53155	MND	MND	MND	MND	110	100	25	20
HP-51210	MND	MND	MND	MND	240	90	80	80
HP-51388	MND	MND	MND	MND	180	200	10	10
HP-49255	MND	MND	MND	MND	300	300	20	20
HP-49244	MND	MND	MND	MND	300	300	30	20
HP-53129	MND	MND	MND	MND	120	115	30	25
HP-53081	MND	MND	MND	MND	280	300	30	45
HP-56294	MND	MND	MND	MND	200	300	NA	25
HP-50671	MND	MND	MND	MND	170	300	80	80
HP-53087	MND	MND	MND	MND	240	140	60	30
HP-53067	MND	MND	MND	MND	140	150	50	20
HP-56298	MND	MND	MND	MND	200	170	80	95
HP-53157	MND	MND	MND	MND	240	170	55	45
HP-53113	MND	MND	MND	MND	170	200	30	40
HP-53133	MND	MND	MND	MND	200	200	10	5
HP-53149	MND	MND	MND	MND	200	200	15	15
HP-53091	MND	MND	MND	MND	200	200	25	20
HP-53125	MND	MND	MND	MND	200	200	35	30
HP-53093	MND	MND	MND	MND	200	200	45	65
HP-50669	MND	MND	MND	MND	240	200	80	60
HP-53123	MND	MND	MND	MND	175	240	15	25
HP-53135	MND	MND	MND	MND	200	240	30	75
HP-53105	MND	MND	MND	MND	240	260	60	30
HP-50661	MND	MND	MND	MND	200	300	30	20
HP-56951	MND	MND	MND	MND	200	300	60	35
HP-53069	MND	MND	MND	MND	300	300	30	25
HP-53071	MND	MND	MND	MND	300	300	30	20
HP-51710	NA	MND	NA	MND	250	160	2	2
HP-51714	MND	MND	MND	MND	110	NA	15	1
HP-56955	MND	MND	MND	MND	Positive	NA	10	NA
HP-58406	NA	NA	NA	NA	NA	NA	5	25
HP-56961	NA	NA	NA	NA	NA	NA	15	5

MND - Mutation not detected; E545X - E545A, G, D, K; Q546X - Q546E, K, R, L; Positive - PTEN positive, unable to determine H-Score; NA - Not available.

### Relationship between Ki67 expression and PI3K pathway status

**Figure 2:**
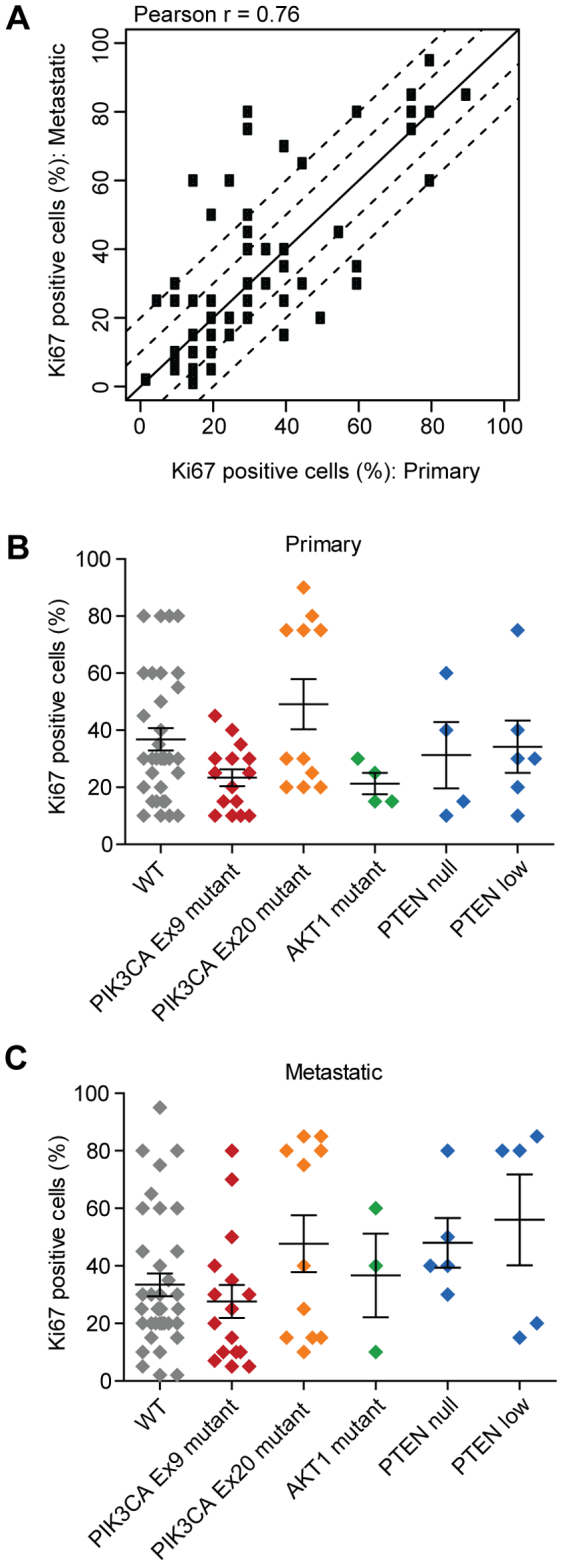
Ki67 status in primary and metastatic samples and relationship to PI3K pathway activation. (A) Correlation between Ki67 staining levels determined by immunohistochemistry in matched primary and metastatic pairs (N = 71). The solid diagonal line (y = x) and the dashed lines (y = x±10, y = x±20) are shown to highlight the magnitude of the absolute differences between x and y axes. (B, C) Ki67 staining levels in primary (B) or metastatic (C) tumors with PI3K pathway alterations. The horizontal lines represent the mean Ki67staining level ± standard error.

IHC for the nuclear antigen Ki67 is a widely used assay for determining relative proliferation rates between tumor samples and has both predictive and prognostic implications [16]. We assessed Ki67 staining via IHC across the collection of paired primary and metastatic samples using criteria recommended by the international Ki67 working group [16]. Overall Ki67 intensity levels were well correlated between primary and metastatic samples (Pearson *r* = 0.76, Figure 2A). *PIK3CA* mutations have in some studies been linked to reduced pathway signaling and better prognosis [17], [18], so we examined whether Ki67 percent positivity was inversely associated with PI3K pathway alterations. We found no significant difference in Ki67 levels in either primary or metastatic tumors when comparing tumors harboring *PIK3CA* mutations, *AKT* mutations, or loss of PTEN expression to tumors with no detected pathway alterations (Figure 2B and 2C). However, primary tumors with *PIK3CA* exon 20 mutations had higher average Ki67 levels than tumors with exon 9 mutations (p = 0.049, Figure 2B), suggesting some subtleties between the two main classes of *PIK3CA* mutations.

### Development of a microfluidic breast cancer gene expression assay

**Figure 3:**
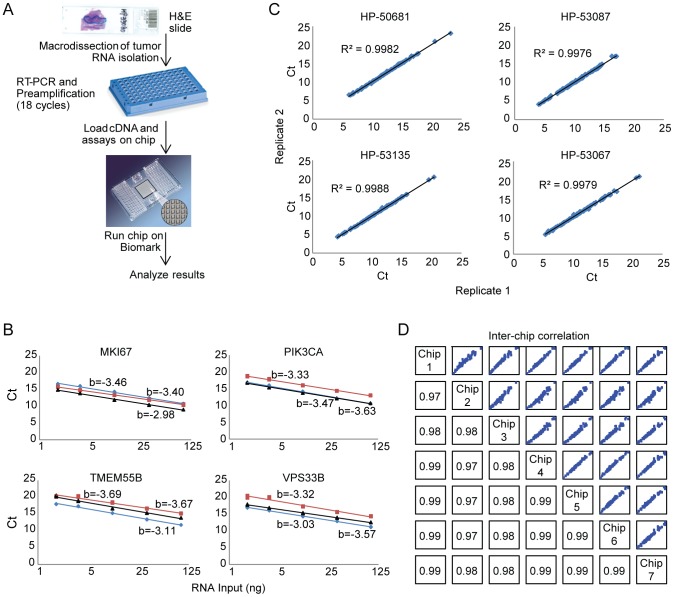
Development of a high-throughput microfluidic gene expression assay for analysis of FFPE breast tumor samples. (A) Schematic of the gene expression assay protocol. (B) Representative five-point standard curves of FFPE tumor RNA (red and black lines) and universal RNA (blue line) run on the breast cancer gene expression assay (slope of line indicated). (C) Intra-chip reproducibility of FFPE tumor samples run on the same 96.96 Dynamic Array. (D) Inter-chip reproducibility of the breast cancer gene expression assay assessed by comparing Ct values of universal RNA across seven independent assay runs. R-squared values are indicated in boxes.

In order to look more broadly at biological changes between primary and metastatic tumors, we developed a gene expression assay based on microfluidic qRT-PCR assessment that works robustly on limited amounts of FFPE tissue and allows for the simultaneous analysis of 90 genes (Figure 3A and Table S2). Content for the panel was based on assessment of genes with differential expression between breast cancer cell lines of known subtypes, published reports of proliferation markers, and genes associated with epithelial-mesenchymal transition and PI3K pathway signaling [9], [19]. The contents of the complete panel are shown in Table S2. Tumor specimens were macrodissected to enrich for tumor cells, and the isolated RNA subjected to preamplification with gene specific primers. Each assay was individually validated for linearity and sensitivity by testing on a range of RNA inputs (10−100 ng) from universal RNA (uRNA) and two FFPE samples (Figure 3B and Fig. S1). All non-reference gene assays were found to have a dynamic range of at least 50-fold across a test set of 30 breast tumor samples (Fig. S2). To further validate the panel, samples were run in duplicate on the same chip and found to show strong intra-chip reproducibility (Figure 3C). Inter-chip reproducibility was assessed by comparing the uRNA Ct values across seven independent chips. Correlation coefficients were at least 0.97 for all chips, and 0.99 for ten of the 21 chip comparisons (Figure 3D).

### Biological validation of the microfluidic breast cancer gene expression assay

**Figure 4:**
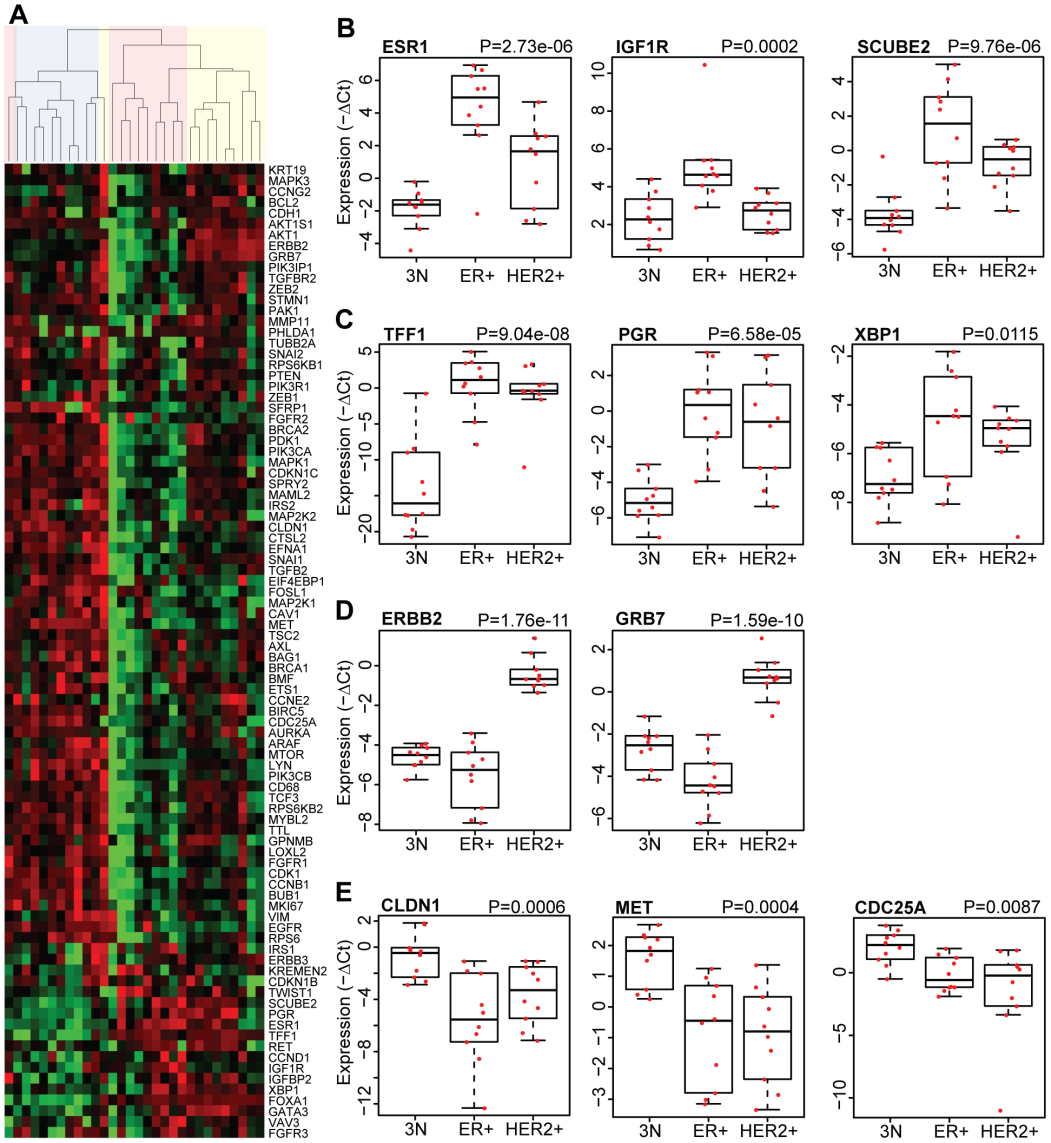
Biological validation of the breast cancer gene expression assay using samples of known immunohistochemical subtype. (A) Hierarchical clustering of thirty FFPE breast cancer tumor samples with known ER, PR and HER2 status run on the breast cancer gene expression assay. Blue =  triple negative, Pink  =  ER+, Yellow = HER2+ (red = high expression, green = low expression) (B) Box-plots indicating genes that showed statistically significant differential expression in the ER+ subtype, (C) ER+ and HER2+ subtype, (D) HER2+ subtype and (E) triple negative subtype samples (3N) (p-values indicated).

As a first step to biologically validate the content of the panel, RNA from a panel of 24 breast cancer cell lines of known molecular subtype [20], [21] was analyzed on the microfluidic gene expression platform. Hierarchical clustering analysis showed that the 90 gene assay organized the cell lines into appropriate luminal and basal-like clusters in all but two cases (Fig. S3). Next, RNA from thirty FFPE samples of known breast cancer subtype based on IHC for ER, PR and HER2 was run on the microfluidic gene expression platform and analyzed by unsupervised hierarchical clustering (Figure 4A). The 90 gene panel was able to correctly cluster all but two samples into the appropriate subtype predicted based on IHC. An F-test was used to identify genes that showed a statistically significant difference between subtypes. *ESR1*, *IGF1R*, *SCUBE2*, *IGFBP2*, *CCND1* and *TWIST1* showed significant association with ER+ status (Figure 4B, Fig. S4). *TFF1*, *PGR*, *XBP1, FOXA1* and *GATA3* were overexpressed in both ER+ and HER2+ tumors, while *ERBB2* and *GRB7* were high specifically in HER2+ tumors (Figure 4C and D, respectively, Fig. S4). Genes enriched in triple negative tumors included *CLDN1*, *MET*, *CDC25A*, *SPRY2*, *SNAI2*, *FOSL1* and *BUB1* (Figure 4E, Fig. S4).

### Application of the gene expression panel to matched primary and metastatic tumor samples

**Figure 5:**
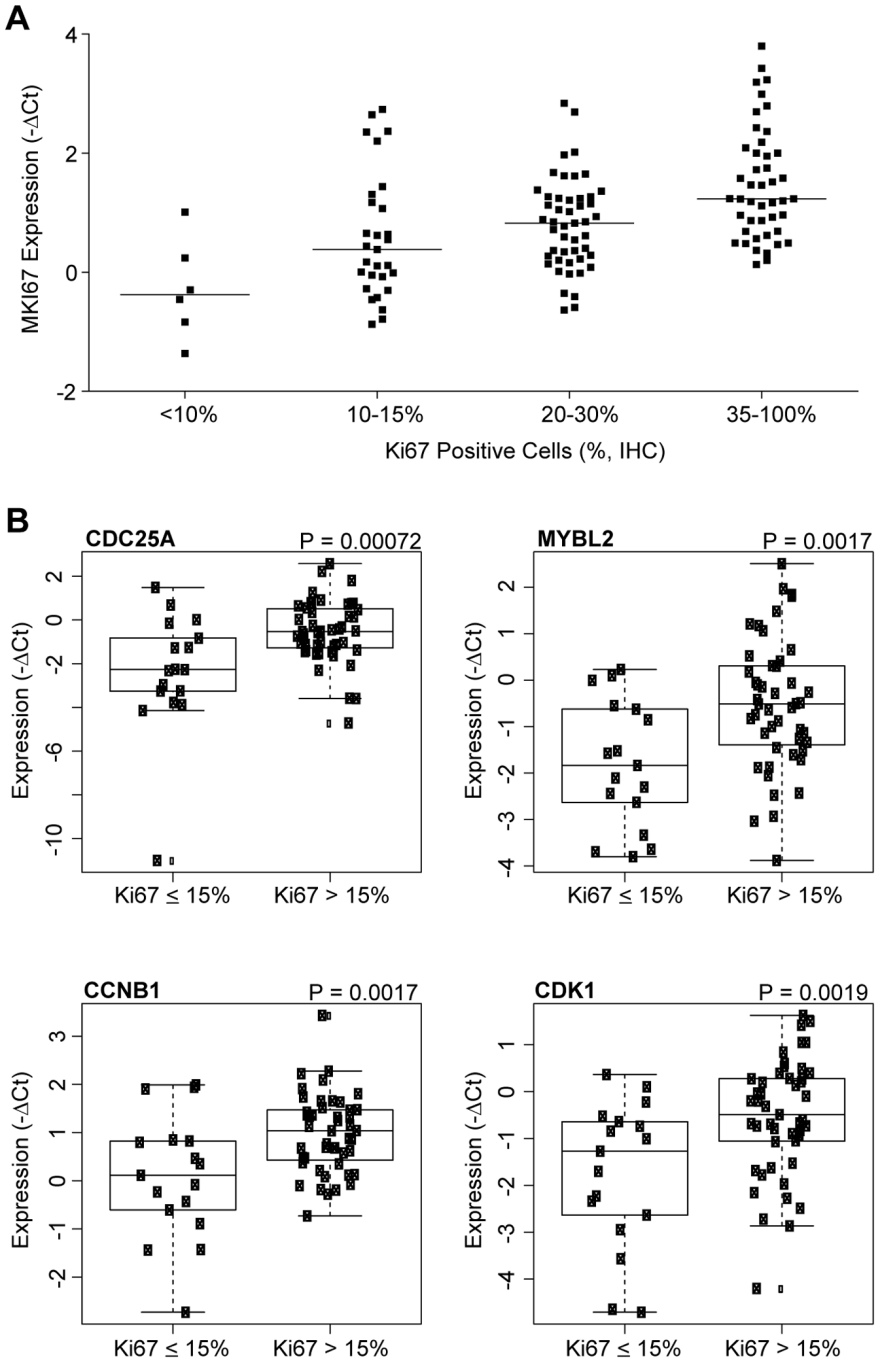
Ki67 protein and gene expression analysis. (A) *MKI67* mRNA expression levels and relationship to Ki67 protein staining levels as determined by IHC. (B) Differentially expressed genes associated with Ki67 high or low protein staining levels (p-values indicated). Ki67 ≤ 15%, N = 16, Ki67 > 15%, N = 48.

RNA from 61 of the matched ER+ tumor samples was analyzed using the microfluidic gene expression panel. As a first step, we compared *MKI67* (the mRNA encoding Ki67 antigen) levels with protein levels determined by IHC (Figure 5A), and found a trend towards higher mRNA expression in tumors with higher nuclear Ki67 protein expression. Analysis of genes differentially expressed between Ki67 high and low primary samples, based on a cutoff of 15% positivity, identified the proliferation genes *CDC25A*, *MYBL2*, *CCNB1* and *CDK1* as showing significantly higher expression in Ki67high primary tumors (Figure 5B).

**Figure 6:**
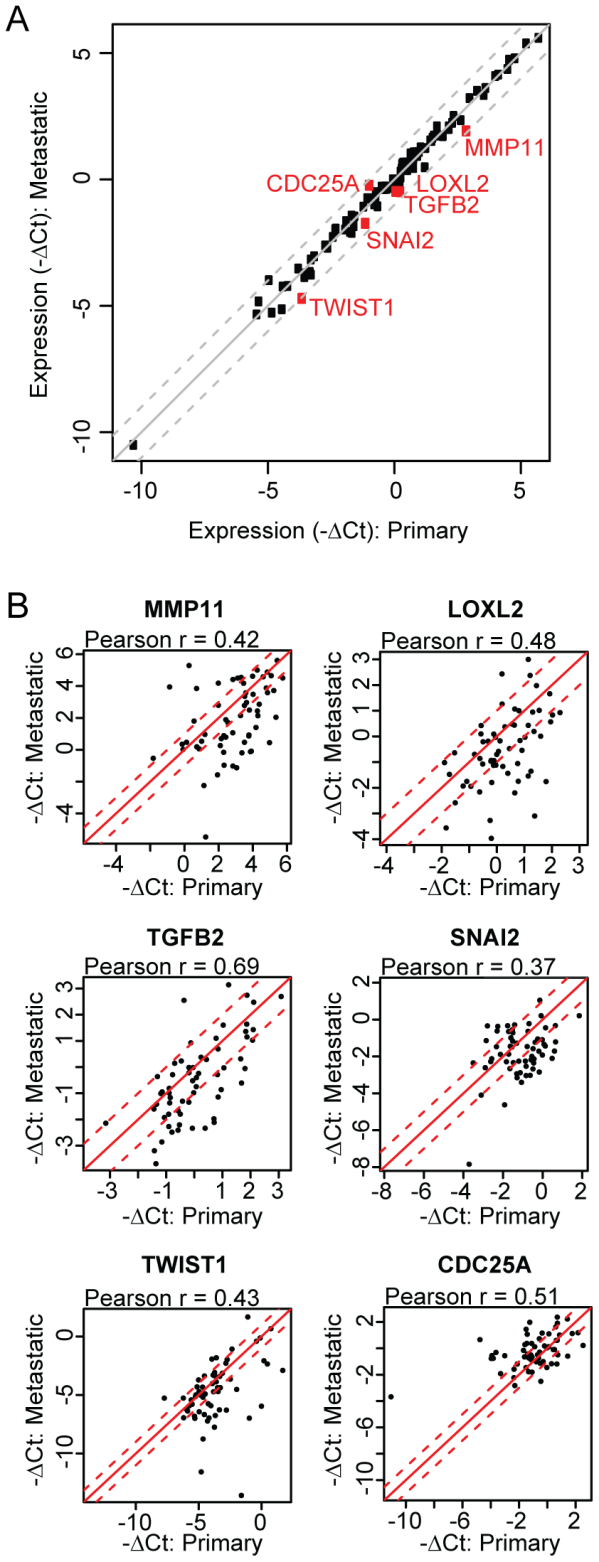
Gene expression correlations between matched primary and metastatic ER+ breast cancer tumor samples. (A) Gene expression correlations between 61 matched primary and metastatic tumor samples for 90 genes from the breast cancer gene expression assay. Each dot represents the mean fold change between primary and metastatic samples for a single gene. (B) Correlation plots of genes that showed a greater than 1.5 fold difference in expression between matched primary and metastatic samples (FDR-adjusted P<0.05). Each dot represents the fold change between primary and metastatic samples for a single patient. The solid diagonal line (y = x) and the dashed lines (y = x±1) are shown to highlight the magnitude of the absolute differences between x and y axes.

We also compared overall gene expression status for the 90 genes between primary and metastatic tumor samples using a paired t-test. This analysis revealed a high level of overall correlation for the 90 gene panel between primary and metastatic samples (Figure 6A). Only six of the 90 genes evaluated showed a statistically significant change of greater than 1.5 fold between primary and metastatic samples (Figure 6B). Several of the genes are implicated in epithelial-mesenchymal transition, including *TWIST1*, *SNAI2* and *TGFB2*. In addition, gene expression between matched primary and metastatic samples showed a significantly higher correlation (median Pearson *r*  =  0.92) than unmatched primary and metastatic samples (median Pearson *r*  =  0.82; Wilcoxon *P* < 0.001).

## Discussion

Estrogen receptor positive breast cancer is a disease that has a particularly long natural history, with more than half of all disease recurrences occurring six years or more after diagnosis, often after treatment with up to five years of adjuvant anti-estrogen therapy [22]. This long time frame could conceivably result in substantial tumor evolution under selective pressure from hormonal therapy, and changes in tumor molecular genetics could confound diagnostic evaluations based on archival tissue intended to guide therapy in the metastatic setting. Such tumor evolution has been extensively documented in matched primary and metastatic renal cell carcinomas [23] and has been examined at the sequence level in triple-negative breast cancer [24], but has not been studied extensively in ER+ breast cancer. Here we set out to understand how a panel of disease-relevant biomarkers changed between primary and metastatic ER+ breast cancer tissues collected a minimum of six months apart, and after adjuvant or front-line metastatic therapy.

The panel of assays included a collection of PI3K pathway alterations, since this pathway appears to play a major cooperative role in ER+ cancers. Numerous studies have shown that *PIK3CA* is the most frequently mutated oncogene in ER+ breast cancer, occurring with a frequency of up to 45% in various tumor series [25], [26], [27]. *PIK3CA* mutations are thought to arise early in cancer development and to be selected for throughout breast cancer progression, as they can be found in ductal carcinoma in situ as well as invasive primary breast cancers and metastatic samples [7]. *PIK3CA* mutations have been associated with a modest negative effect on responsiveness to endocrine therapy in several neoadjuvant studies [28]. One model would be that acquisition of *PIK3CA* mutations contributes to resistance to such therapies and would be expected to show higher prevalence in samples collected after disease recurrence. Indeed, a previous study showed *PIK3CA* mutations may be discordant between primary and metastatic breast cancer, with a higher prevalence in metastatic samples [29]. In this study of 73 paired samples with data for both primary and metastatic samples, we identified five pairs with a mutation in metastatic tissue but not the primary tumor, and two samples with a mutation in the primary tumor but not the metastatic sample. Thus, acquisition of *PIK3CA* mutations does not appear to be a widespread resistance mechanism following hormonal and chemotherapy in the adjuvant setting. We also found activating mutations in *AKT1* in a small percentage of cases, as has been reported previously [30]. Consistent with other recent reports [6], [7], our results suggest that discordance between *PIK3CA* and *AKT1* mutations in asynchronous samples may be due to heterogeneity in the primary cancer, where metastatic clones may be derived from either mutant or non-mutant progenitor cells, since discordance can occur in either direction.

Our studies suggest a lower prevalence of PTEN loss than has recently been reported in other ER+ cohorts [6], between five and ten percent as opposed to nearly 30%. These findings could be explained by differences in the IHC assay or the patient population, but are also consistent with previous reports suggesting PTEN loss is more predominant in triple-negative breast cancer than hormone receptor positive disease [27], [31]. This finding may have implications for clinical trials attempting to validate PTEN as a diagnostic marker for PI3K targeting therapies in ER+ breast cancer. Notably, alterations in *PIK3CA*, PTEN and *AKT1* were generally non-overlapping, suggesting perhaps that they are functionally redundant in activating this signaling pathway.

Unlike previous reports, we also addressed whether biomarker alterations in the PI3K pathway were associated with a reduced proliferation state, given previous studies suggesting that *PIK3CA* mutations are associated with low pathway output [17], [18] and favorable prognosis [32]. Importantly, we found Ki67 levels to be independent of *PIK3CA* mutation status or other alterations in the pathway, suggesting that selecting cohorts of patients based on pathway activation would not necessarily identify low risk patients with non-proliferative cancers. We did observe higher proliferation rates in exon 20 *PIK3CA* mutant cancers, consistent with previous reports showing differences in prognostic value between exon 9 and exon 20 mutations, though sometimes in opposing directions [33], [34]. The observed differences suggest these alterations should perhaps be evaluated independently in the context of predicting response to targeted therapies.

Gene expression predictors of risk of recurrence have shown promise in early breast cancer, and are widely used to make therapeutic decisions (ie. hormonal versus chemotherapy) based on relative risk assessment [35]. As of yet, gene expression based assays have not seen widespread incorporation as predictive diagnostic tools in metastatic disease. Recent studies have suggested such signatures could have promise as a general method of determining the overall state of pathway activation [17] or predicting response to targeted therapies [9], a conceptually appealing approach in that it may cast a wider net and identify patients who would be missed by single-analyte assays. Multiplex gene expression assays could also have applications in confirming molecular subtype, determination of epithelial-mesenchymal phenotype, or identifying more highly proliferative tumors. However, collection of fresh biopsies in patients with metastatic disease is not feasible in many cases, so clinical application of this technology requires demonstrating that molecular portraits inferred from primary, archival tumor material can give a reasonable facsimile of later metastatic disease. We have developed a microfluidic gene expression assay that retains the sensitivity and dynamic range of qRT-PCR and allows simultaneous evaluation of 90 genes relevant to breast cancer subtyping, proliferation, epithelial-mesenchymal biology and PI3K pathway signaling from small amounts of RNA extracted from FFPE tissue. Unsupervised hierarchical clustering analysis of procured tumor samples of known IHC subtype suggested this assay can efficiently group breast tumor samples into the three major subtypes of hormone receptor positive, HER2+ and triple negative breast cancer. Consistent with expectations, canonical luminal genes such as *ESR1*, *GATA3*, *FOXA1*, *TFF1* and *IGF1R* were higher in ER+ samples compared to triple negative samples, while proliferation and mesenchymal genes such as *SPRY2*, *SNAI2*, *FOSL1*, *MET* and *BUB1* were higher in triple negative cancers. Importantly, analysis of the matched primary and metastatic ER+ breast tumor samples with the microfluidic gene expression assay suggested a generally high degree of concordance, with only six of 90 genes showing more than a 1.5 fold difference between matched primary and metastatic tissue and very high intra-patient correlation between primary and metastatic tumors. Intriguingly, the genes differentially expressed between matched tumor pairs included *TWIST* and *SNAIL2*, which have a well-documented role in metastasis via regulating E-cadherin expression and epithelial-mesenchymal transition [36], [37]. Surprisingly, these genes were more highly expressed in primary relative to metastatic samples, suggesting perhaps that these genes are upregulated in the premetastatic state. We also examined the potential of the gene expression panel to identify more proliferative tumors, as tumors with a higher proliferation index may respond differentially to targeted therapies. Since the majority of samples in this study showed greater than 15% Ki67, the sample set was likely biased towards more proliferative, luminal B type tumors [38]. Nevertheless, we saw excellent general agreement between Ki67 staining by IHC and *MKI67* mRNA levels determined by the microfluidic platform. Moreover, Ki67 high tumors showed higher expression of proliferation genes such as *CDC25A*, *CCNB1* and *CDK1*, suggesting possible utility in identifying tumors with greater proliferative potential.

Over the past 10-15 years, a wealth of information has emerged on the molecular portraits and genomic architecture of breast cancers [25], [26], [27], [39]. However, routine clinical assessment of molecular features of breast cancers in patients with metastatic cancer has lagged behind due to challenges in assessing molecular changes in fixed archival tissues. Here we describe a panel of assays that has been optimized for fixed tissue and which allows reasonably comprehensive assessment of a range of biological pathways and processes. The microfluidic gene expression assay has broad content selected from the collective literature of breast cancer biology and subtypes, and has potential applications to characterize patient samples based on signaling pathway status and biological processes such as EMT. Moreover, the overall platform of assays we describe in this study could have considerable impact on identifying patient subsets responsive to therapeutics targeting the PI3K/mTOR axis. Careful application of the panel of assays to clinical samples may yield answers to questions such as whether clinical benefit is associated with a specific pathway alteration (i.e. *PIK3CA* mutations compared to PTEN), whether patients with more proliferative tumors show differential benefit to such agents, and the role of gene signatures in predicting response and resistance to these agents. The outcome of these trials is eagerly anticipated in the field.

## Supporting Information

Figure S1 Five-point standard curves of FFPE tumor RNA (blue and yellow lines) and universal RNA (red line) run on the breast cancer gene expression assay (slope of line indicated).
(PDF)

Figure S2 Negative delta Ct values of all assays on panel. 25^th^ percentile to 75^th^ percentile indicated at the bottom of each graph.
(PDF)

Figure S3 Hierarchical clustering of 24 breast cancer cell line samples with known molecular subtypes. Black  =  basal-like, Red  =  Luminal (scale: −5 to 5 −ΔCt).
(PDF)

Figure S4 Biological validation of the breast cancer gene expression assay using samples of known immunohistochemical subtype. Box-plots indicating genes that showed statistically significant differential expression in the ER+ subtype, ER+ and HER2+ subtype and triple negative subtype samples (3N) (p-values indicated).
(PDF)

Table S1 Patient characteristics and treatment information.
(XLSX)

Table S2 Breast cancer gene expression assay content.
(XLSX)
