# Supplementary figures and images for: Targeted Biomarker Profiling of Matched Primary and Metastatic Estrogen Receptor Positive Breast Cancers
Source: PLoS One. 2014 Feb 10;9(2):e88401. doi: 10.1371/journal.pone.0088401 (PMC3919784; doi:10.1371/journal.pone.0088401)

Supplemental Figure S1

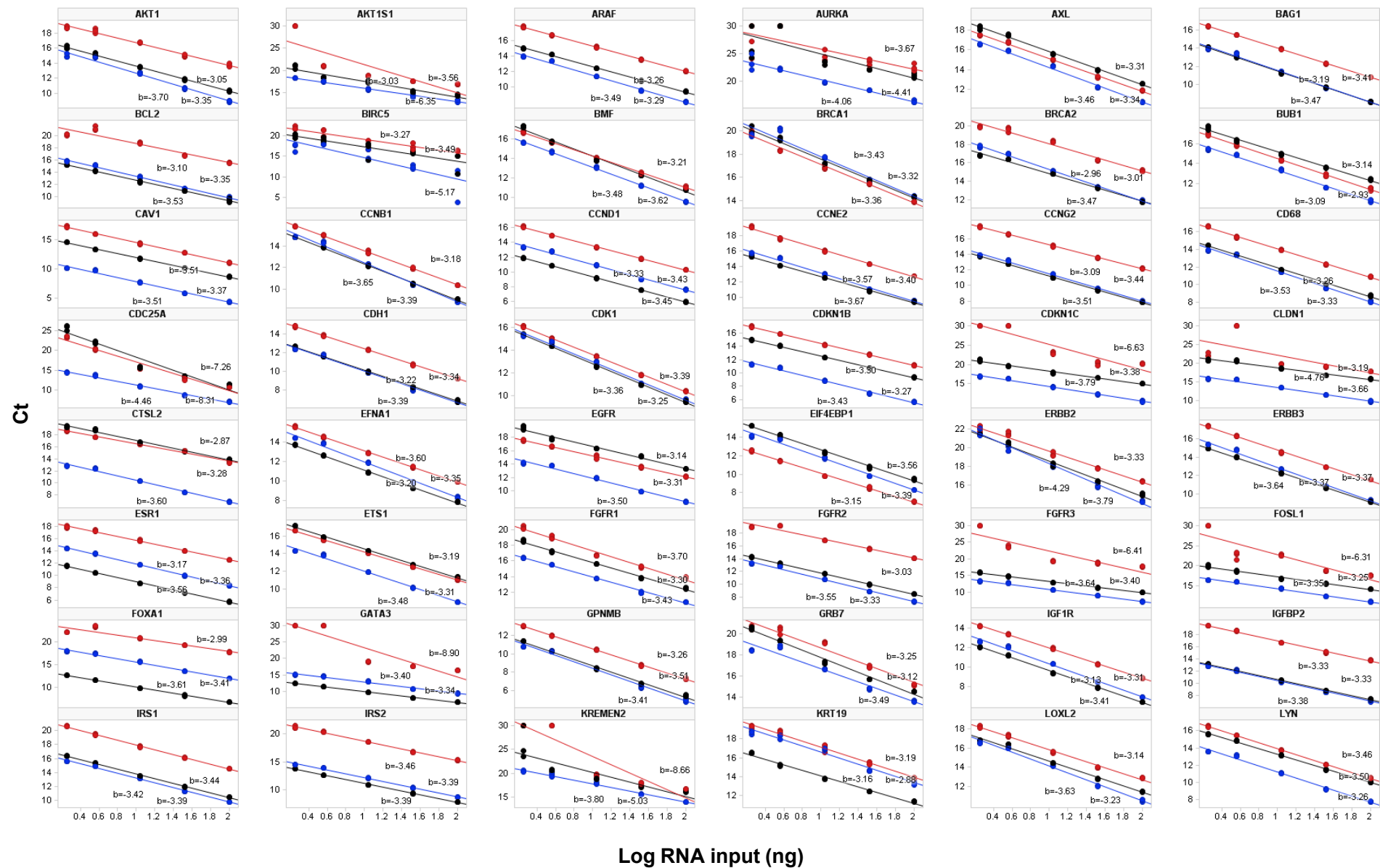

Ct

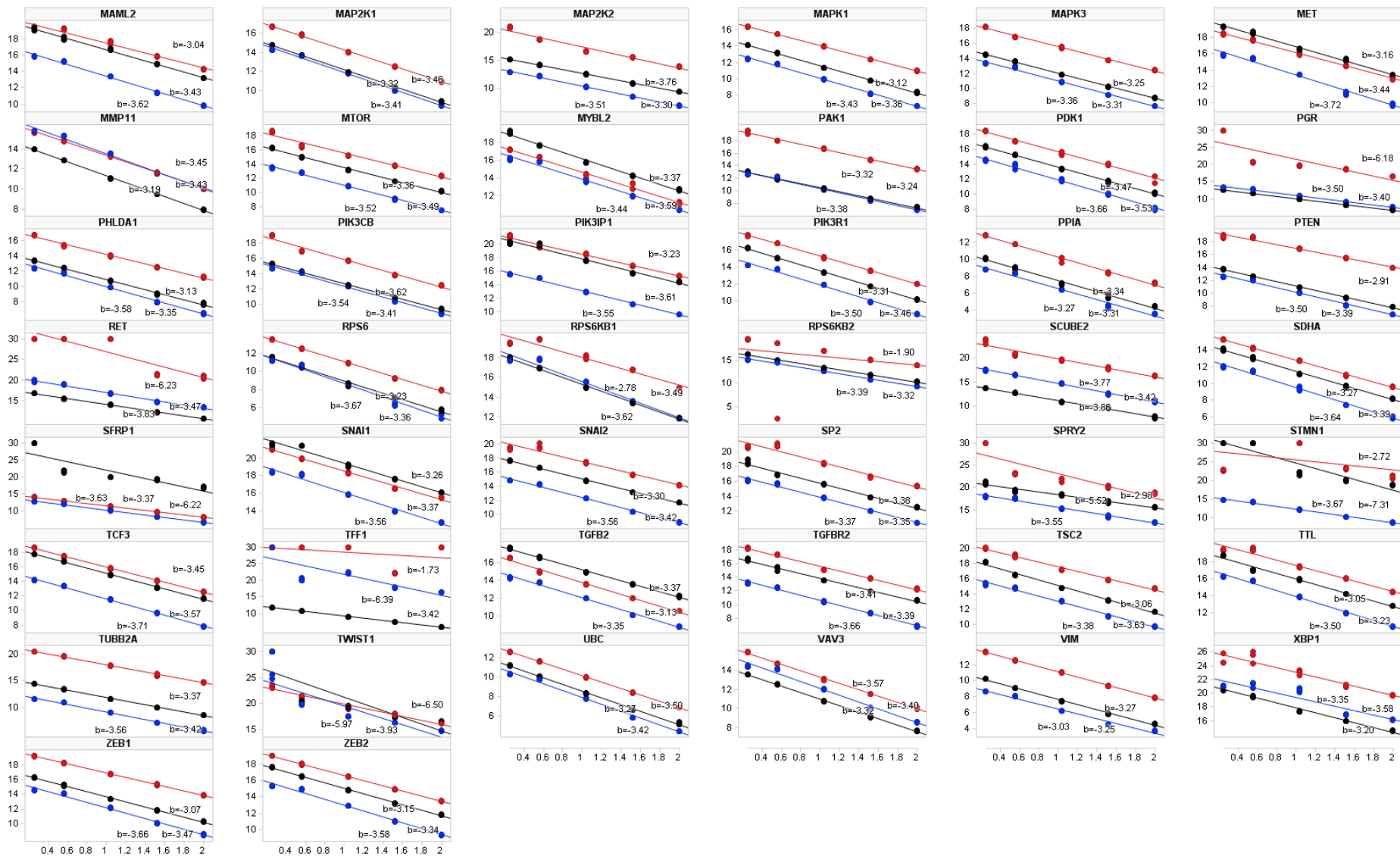

Log RNA input (ng)

Supplement: Figure S1 — Five-point standard curves of FFPE tumor RNA (blue and yellow lines) and universal RNA (red line) run on the breast cancer gene expression assay (slope of line indicated). (PDF) [file pone.0088401.s001.pdf]

Supplemental Figure S2

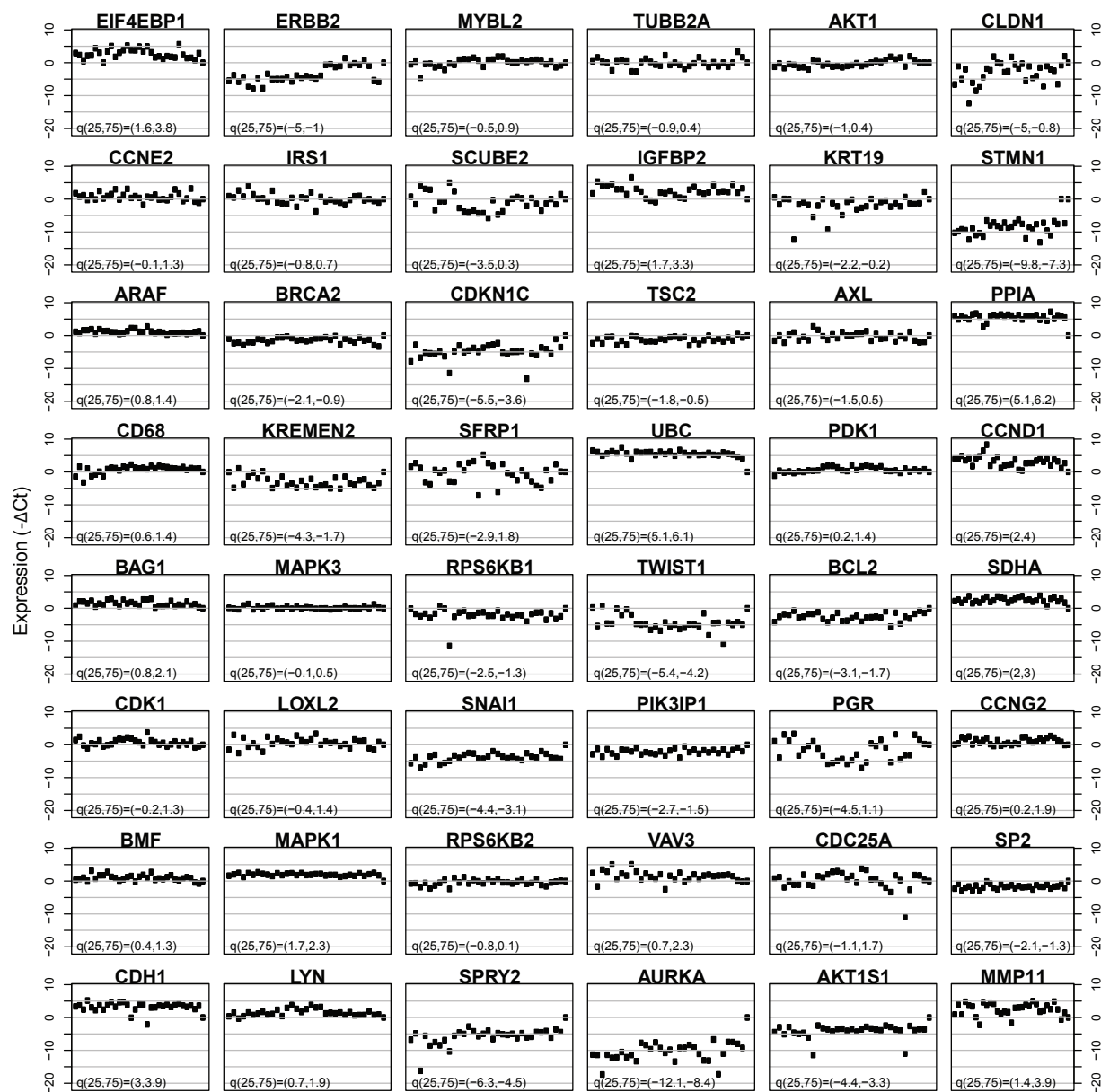

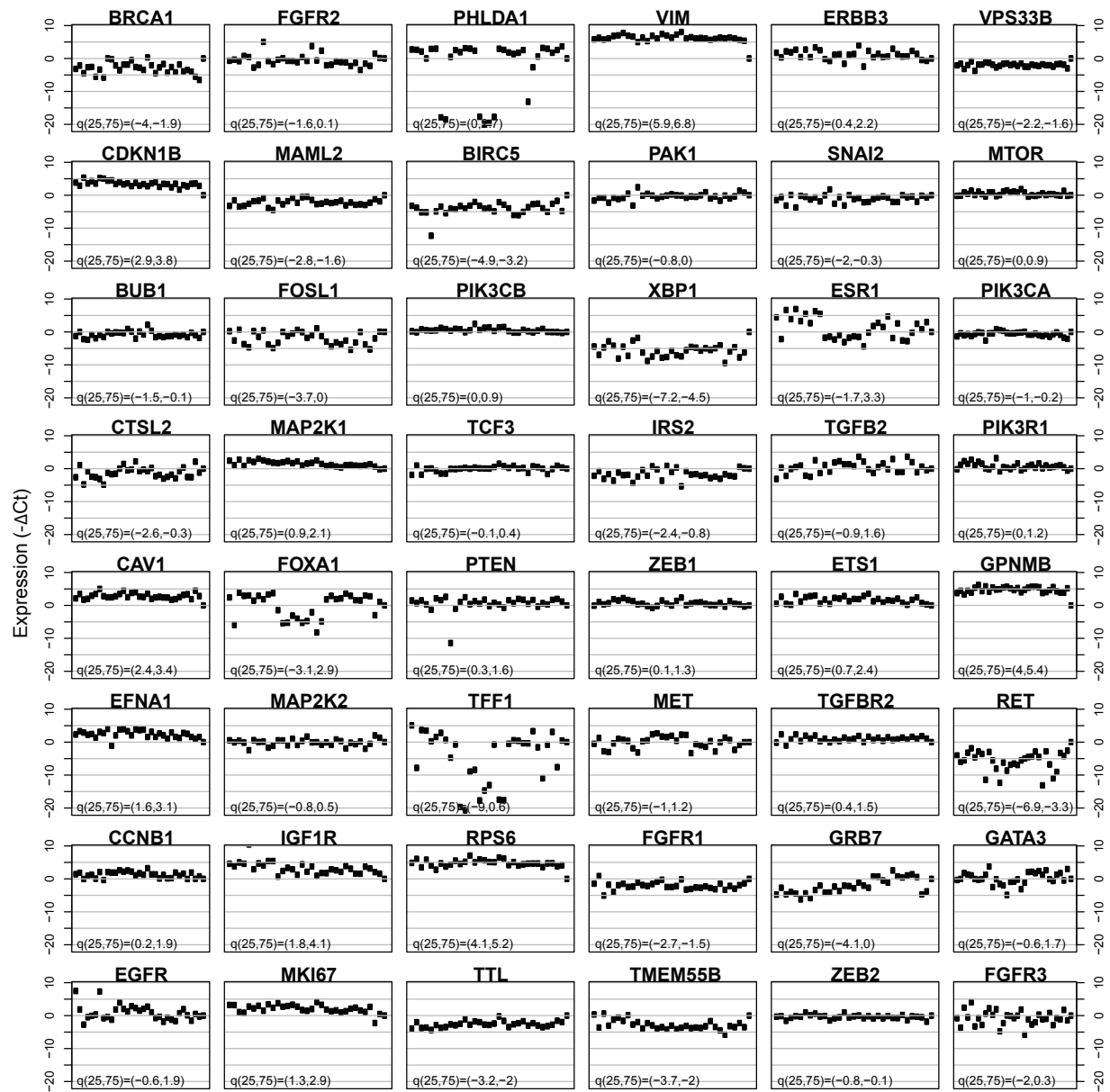

Supplement: Figure S2 — Negative delta Ct values of all assays on panel. 25th percentile to 75th percentile indicated at the bottom of each graph. (PDF) [file pone.0088401.s002.pdf]

Supplemental Figure S3

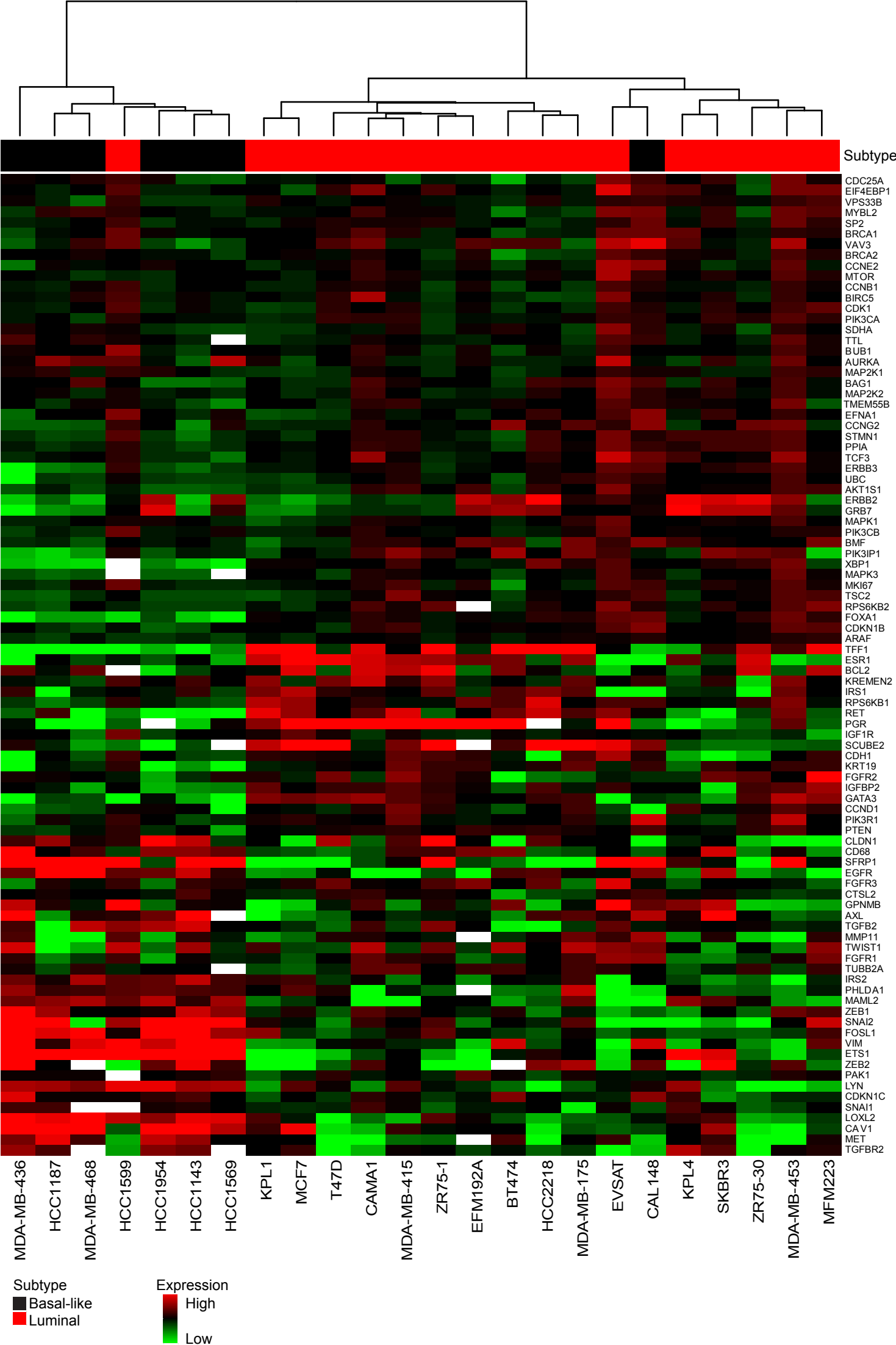

Supplement: Figure S3 — Hierarchical clustering of 24 breast cancer cell line samples with known molecular subtypes. Black = basal-like, Red = Luminal (scale: −5 to 5 −ΔCt). (PDF) [file pone.0088401.s003.pdf]

Supplemental Figure S4

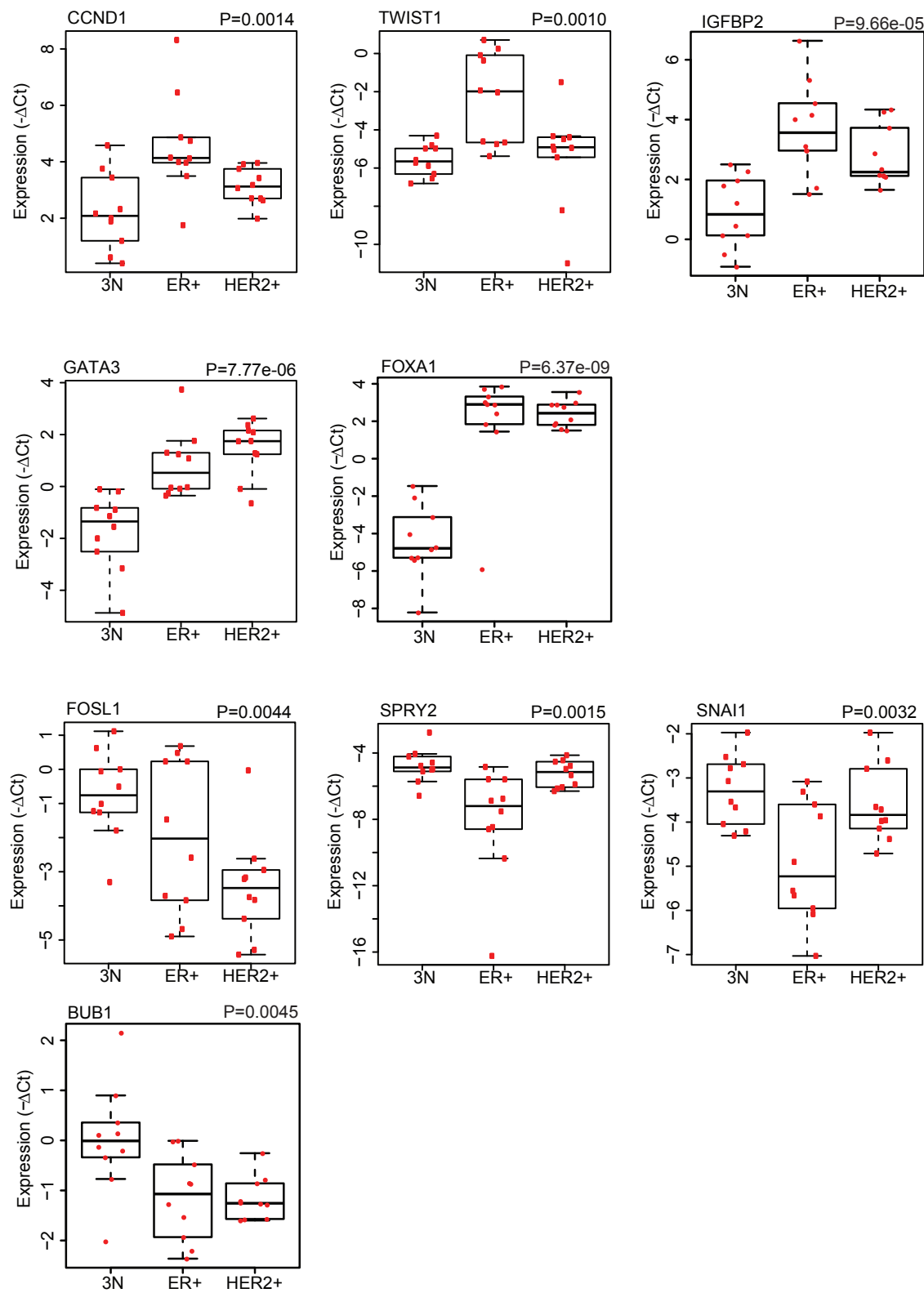

Supplement: Figure S4 — Biological validation of the breast cancer gene expression assay using samples of known immunohistochemical subtype. Box-plots indicating genes that showed statistically significant differential expression in the ER+ subtype, ER+ and HER2+ subtype and triple negative subtype samples (3N) (p-values indicated). (PDF) [file pone.0088401.s004.pdf]
